# Hexaaqua­cobalt(II) bis­[4-(pyridin-2-yl­meth­oxy)benzoate] dihydrate

**DOI:** 10.1107/S1600536811040931

**Published:** 2011-10-12

**Authors:** Li-Wei Zhang, Shan Gao, Seik Weng Ng

**Affiliations:** a Key Laboratory of Functional Inorganic Material Chemistry, Ministry of Education, Heilongjiang University, Harbin 150080, People’s Republic of China; b Department of Chemistry, University of Malaya, 50603 Kuala Lumpur, Malaysia; c Chemistry Department, Faculty of Science, King Abdulaziz University, PO Box 80203 Jeddah, Saudi Arabia

## Abstract

The Co^II^ atom in the title salt, [Co(H_2_O)_6_](C_13_H_10_NO_3_)_2_·2H_2_O, lies on a center of inversion in an octa­hedron of water mol­ecules. The cations, anions and uncoordinated water mol­ecules are linked by O—H⋯O and O—H⋯N hydrogen bonds into a three-dimensional network. The anion is essentially planar, with an r.m.s. deviation of all non-H atoms of 0.066 Å.

## Related literature

There are many examples of hexa­aqua­cobalt(II) benzoates; these benzoates possess substitutents capable of serving as hydrogen-bond acceptors/donors, see: Deng *et al.* (2006 ▶). For the isotypic Mn(II) salt, see: Zhang *et al.* (2011 ▶).
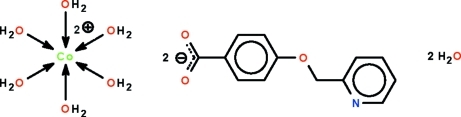


## Experimental

### 

#### Crystal data

[Co(H_2_O)_6_](C_13_H_10_NO_3_)_2_·2H_2_O
*M* *_r_* = 659.50
Triclinic, 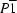

*a* = 7.4349 (3) Å
*b* = 7.5431 (3) Å
*c* = 13.7531 (6) Å
α = 84.307 (1)°
β = 83.153 (1)°
γ = 73.333 (1)°
*V* = 731.91 (5) Å^3^
*Z* = 1
Mo *K*α radiation
μ = 0.66 mm^−1^
*T* = 293 K
0.21 × 0.15 × 0.13 mm

#### Data collection

Rigaku R-AXIS RAPID IP diffractometer
Absorption correction: multi-scan (*ABSCOR*; Higashi, 1995 ▶) *T*_min_ = 0.874, *T*_max_ = 0.919
7251 measured reflections
3314 independent reflections
2401 reflections with *I* > 2σ(*I*)
*R*_int_ = 0.026

#### Refinement

*R*[*F* ^2^ > 2σ(*F* ^2^)] = 0.036
*wR*(*F* ^2^) = 0.138
*S* = 1.15
3314 reflections
228 parameters
18 restraints
H atoms treated by a mixture of independent and constrained refinement
Δρ_max_ = 0.63 e Å^−3^
Δρ_min_ = −0.69 e Å^−3^

### 

Data collection: *RAPID-AUTO* (Rigaku, 1998 ▶); cell refinement: *RAPID-AUTO*; data reduction: *CrystalClear* (Rigaku/MSC, 2002 ▶); program(s) used to solve structure: *SHELXS97* (Sheldrick, 2008 ▶); program(s) used to refine structure: *SHELXL97* (Sheldrick, 2008 ▶); molecular graphics: *X-SEED* (Barbour, 2001 ▶); software used to prepare material for publication: *publCIF* (Westrip, 2010 ▶).

## Supplementary Material

Crystal structure: contains datablock(s) global, I. DOI: 10.1107/S1600536811040931/bt5664sup1.cif

Structure factors: contains datablock(s) I. DOI: 10.1107/S1600536811040931/bt5664Isup2.hkl

Additional supplementary materials:  crystallographic information; 3D view; checkCIF report

## Figures and Tables

**Table 1:** Hydrogen-bond geometry (Å, °)

*D*—H⋯*A*	*D*—H	H⋯*A*	*D*⋯*A*	*D*—H⋯*A*
O1w—H11⋯O1	0.84 (1)	1.94 (1)	2.762 (3)	169 (3)
O1w—H12⋯O4w^i^	0.84 (1)	1.87 (1)	2.696 (4)	171 (4)
O2w—H21⋯O2	0.84 (1)	1.84 (1)	2.678 (3)	179 (3)
O2w—H22⋯O2^ii^	0.84 (1)	1.97 (2)	2.755 (3)	156 (3)
O3w—H31⋯O1^iii^	0.85 (1)	1.96 (1)	2.798 (3)	170 (4)
O3w—H32⋯N1^iv^	0.85 (1)	1.95 (1)	2.799 (3)	177 (4)
O4w—H41⋯O2	0.84 (1)	2.22 (4)	2.891 (4)	137 (5)
O4w—H42⋯O3w^v^	0.84 (1)	2.42 (4)	3.176 (4)	151 (7)

Symmetry codes: (i) 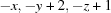; (ii) 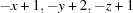; (iii) 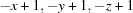; (iv) 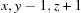; (v) 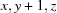.

**Fig. 1.**
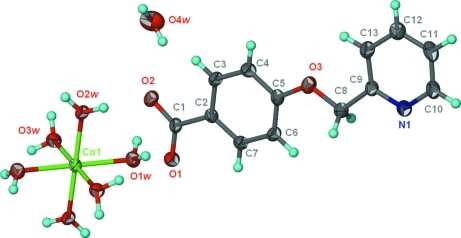
Anisotropic displacement ellipsoid plot (Barbour, 2001) of Co(H2O)6.2(C13H10NO3).2H2O at the 50% probability level; hydrogen atoms are drawn as spheres of arbitrary radius.

## supplementary crystallographic information

### Comment

First-row transition metal dications form a plethora of metal dicarboxylates; however, occasionally, no direct metal–carboxylate bond is formed, and the product consists of hexaaquametal cations and carboxylate ions, the anion interacting indirectly in an outer-sphere type of coordination. 4-(Pyridin-2-ylmethoxy)benzoic acid is a commercially available carboxylic acid but there are no reports on its metal carboxylates. The reaction of the deprotonated acid with cobalt(II) ions gives the hexaaquacobalt(II) salt (Scheme I, Fig. 1). The Co^II^ atom in the salt lies on a center-of-inversion in an octahedron of water molecules. The metal atom interacts with the carboxylate ion indirectly, through the coordinated water molecules, in an outer-sphere type of coordination. The cations, anions and lattice water molecules are linked by O···H···O and O–H···N hydrogen bonds into a three-dimensional network (Table 1).

### Experimental

Cobalt nitrate (1 mmol) was added to an aqueous solution of 4-(pyridin-2-ylmethoxy)benzoic acid (2 mmol) that was earlier been treated with 1*M* sodium hydroxide to a pH of 6. The filtered solution was set aside for several days, after which pink prismatic crystals separated from solution.

### Refinement

Carbon-bound H-atoms were placed in calculated positions (C–H 0.93 Å) and were included in the refinement in the riding model approximation, with *U*(H) set to 1.2*U*(C). The water H-atoms were located in a difference Fourier map, and were refined with distance restraints of O–H 0.84±0.01 Å and H···H 1.37±0.01 Å; their displacement factors were refined.

The anisotropic displacement ellipsoids of the lattice water O were restrained to an isotropic behaviour.

The (2 2 7) reflection was omitted owing to bad agreement.

### Crystal data

**Table:**

[Co(H_2_O)_6_](C_13_H_10_NO_3_)_2_·2H_2_O	*Z* = 1
*M**_r_* = 659.50	*F*(000) = 345
Triclinic, *P*1	*D*_x_ = 1.496 Mg m^−^^3^
Hall symbol: -P 1	Mo *K*α radiation, λ = 0.71073 Å
*a* = 7.4349 (3) Å	Cell parameters from 5547 reflections
*b* = 7.5431 (3) Å	θ = 3.1–27.4°
*c* = 13.7531 (6) Å	µ = 0.66 mm^−^^1^
α = 84.307 (1)°	*T* = 293 K
β = 83.153 (1)°	Prism, pink
γ = 73.333 (1)°	0.21 × 0.15 × 0.13 mm
*V* = 731.91 (5) Å^3^	

### Data collection

**Table:**

Rigaku R-AXIS RAPID IP diffractometer	3314 independent reflections
Radiation source: fine-focus sealed tube	2401 reflections with *I* > 2σ(*I*)
graphite	*R*_int_ = 0.026
ω scans	θ_max_ = 27.4°, θ_min_ = 3.1°
Absorption correction: multi-scan (*ABSCOR*; Higashi, 1995)	*h* = −9→9
*T*_min_ = 0.874, *T*_max_ = 0.919	*k* = −9→9
7251 measured reflections	*l* = −17→17

### Refinement

**Table:**

Refinement on *F*^2^	Primary atom site location: structure-invariant direct methods
Least-squares matrix: full	Secondary atom site location: difference Fourier map
*R*[*F*^2^ > 2σ(*F*^2^)] = 0.036	Hydrogen site location: inferred from neighbouring sites
*wR*(*F*^2^) = 0.138	H atoms treated by a mixture of independent and constrained refinement
*S* = 1.15	*w* = 1/[σ^2^(*F*_o_^2^) + (0.0658*P*)^2^ + 0.3695*P*] where *P* = (*F*_o_^2^ + 2*F*_c_^2^)/3
3314 reflections	(Δ/σ)_max_ = 0.001
228 parameters	Δρ_max_ = 0.63 e Å^−^^3^
18 restraints	Δρ_min_ = −0.69 e Å^−^^3^

### Fractional atomic coordinates and isotropic or equivalent isotropic displacement parameters (Å^2^)

**Table:**

	*x*	*y*	*z*	*U*_iso_*/*U*_eq_	
Co1	0.5000	0.5000	0.5000	0.02876 (18)	
O1	0.4152 (3)	0.8175 (3)	0.26860 (15)	0.0429 (5)	
O2	0.3638 (3)	1.0264 (3)	0.37899 (14)	0.0415 (5)	
O3	0.2477 (4)	1.5730 (3)	−0.00100 (15)	0.0493 (6)	
O1W	0.2738 (3)	0.5989 (3)	0.41333 (15)	0.0368 (5)	
O2W	0.4601 (3)	0.7721 (2)	0.52791 (14)	0.0368 (5)	
O3W	0.3196 (3)	0.4485 (3)	0.62650 (14)	0.0376 (5)	
O4W	0.0843 (4)	1.2664 (5)	0.5083 (3)	0.0673 (8)	
N1	0.1751 (4)	1.7407 (3)	−0.25030 (18)	0.0403 (6)	
C1	0.3773 (4)	0.9820 (4)	0.2913 (2)	0.0321 (6)	
C2	0.3421 (4)	1.1356 (4)	0.21207 (19)	0.0301 (6)	
C3	0.3111 (5)	1.3191 (4)	0.2343 (2)	0.0373 (7)	
H3	0.3107	1.3462	0.2989	0.045*	
C4	0.2809 (5)	1.4610 (4)	0.1611 (2)	0.0403 (7)	
H4	0.2604	1.5830	0.1764	0.048*	
C5	0.2812 (4)	1.4210 (4)	0.0650 (2)	0.0345 (6)	
C6	0.3114 (5)	1.2396 (4)	0.0414 (2)	0.0366 (7)	
H6	0.3109	1.2130	−0.0232	0.044*	
C7	0.3425 (4)	1.0985 (4)	0.1154 (2)	0.0338 (6)	
H7	0.3641	0.9765	0.0998	0.041*	
C8	0.2360 (5)	1.5490 (4)	−0.1010 (2)	0.0363 (7)	
H8A	0.1397	1.4877	−0.1062	0.044*	
H8B	0.3557	1.4734	−0.1294	0.044*	
C9	0.1869 (4)	1.7387 (4)	−0.15412 (19)	0.0317 (6)	
C10	0.1258 (5)	1.9066 (5)	−0.3007 (2)	0.0452 (8)	
H10	0.1169	1.9094	−0.3678	0.054*	
C11	0.0878 (5)	2.0720 (5)	−0.2581 (3)	0.0490 (8)	
H11A	0.0534	2.1840	−0.2955	0.059*	
C12	0.1014 (5)	2.0687 (5)	−0.1596 (3)	0.0502 (8)	
H12A	0.0765	2.1787	−0.1288	0.060*	
C13	0.1528 (5)	1.8992 (4)	−0.1062 (2)	0.0434 (7)	
H13	0.1642	1.8936	−0.0393	0.052*	
H11	0.304 (5)	0.677 (4)	0.372 (2)	0.065 (13)*	
H12	0.167 (3)	0.646 (5)	0.442 (3)	0.069 (14)*	
H21	0.430 (5)	0.853 (3)	0.4814 (16)	0.048 (10)*	
H22	0.503 (5)	0.816 (4)	0.5708 (17)	0.053 (11)*	
H31	0.390 (5)	0.361 (3)	0.660 (2)	0.065 (12)*	
H32	0.271 (5)	0.536 (3)	0.664 (2)	0.065 (12)*	
H41	0.117 (7)	1.227 (7)	0.4522 (18)	0.10 (2)*	
H42	0.149 (10)	1.336 (11)	0.518 (5)	0.24 (5)*	

### Atomic displacement parameters (Å^2^)

**Table:**

	*U*^11^	*U*^22^	*U*^33^	*U*^12^	*U*^13^	*U*^23^
Co1	0.0379 (3)	0.0210 (3)	0.0273 (3)	−0.0075 (2)	−0.0068 (2)	0.00113 (19)
O1	0.0675 (15)	0.0234 (9)	0.0361 (11)	−0.0079 (10)	−0.0129 (10)	0.0013 (8)
O2	0.0683 (15)	0.0294 (10)	0.0284 (10)	−0.0143 (10)	−0.0137 (10)	0.0032 (8)
O3	0.0880 (18)	0.0294 (10)	0.0294 (11)	−0.0134 (11)	−0.0167 (11)	0.0073 (8)
O1W	0.0426 (13)	0.0308 (10)	0.0367 (11)	−0.0092 (9)	−0.0095 (10)	0.0021 (9)
O2W	0.0612 (14)	0.0230 (9)	0.0277 (10)	−0.0109 (9)	−0.0136 (9)	−0.0005 (8)
O3W	0.0462 (13)	0.0312 (10)	0.0294 (10)	−0.0035 (9)	−0.0002 (9)	0.0000 (9)
O4W	0.0531 (17)	0.0683 (18)	0.077 (2)	−0.0156 (14)	0.0123 (15)	−0.0117 (16)
N1	0.0455 (15)	0.0375 (13)	0.0312 (12)	−0.0026 (11)	−0.0027 (11)	0.0020 (10)
C1	0.0416 (17)	0.0266 (13)	0.0296 (14)	−0.0098 (12)	−0.0121 (12)	0.0026 (11)
C2	0.0354 (15)	0.0248 (12)	0.0294 (13)	−0.0071 (11)	−0.0063 (11)	0.0022 (10)
C3	0.0537 (19)	0.0300 (14)	0.0274 (13)	−0.0080 (13)	−0.0094 (13)	−0.0021 (11)
C4	0.060 (2)	0.0231 (13)	0.0366 (15)	−0.0077 (13)	−0.0107 (14)	0.0000 (11)
C5	0.0428 (17)	0.0305 (14)	0.0286 (13)	−0.0084 (12)	−0.0074 (12)	0.0057 (11)
C6	0.0532 (19)	0.0309 (14)	0.0244 (13)	−0.0088 (13)	−0.0065 (12)	−0.0016 (11)
C7	0.0404 (16)	0.0253 (13)	0.0336 (15)	−0.0044 (12)	−0.0067 (12)	−0.0026 (11)
C8	0.0474 (18)	0.0315 (14)	0.0273 (13)	−0.0076 (13)	−0.0042 (12)	0.0019 (11)
C9	0.0348 (15)	0.0329 (14)	0.0251 (13)	−0.0078 (12)	−0.0024 (11)	0.0040 (11)
C10	0.0486 (19)	0.0471 (18)	0.0302 (15)	−0.0013 (15)	−0.0044 (13)	0.0087 (13)
C11	0.058 (2)	0.0365 (16)	0.0501 (19)	−0.0103 (15)	−0.0161 (16)	0.0150 (14)
C12	0.071 (2)	0.0336 (15)	0.0473 (19)	−0.0124 (16)	−0.0194 (17)	0.0016 (14)
C13	0.060 (2)	0.0358 (15)	0.0352 (15)	−0.0120 (15)	−0.0145 (14)	0.0008 (12)

### Geometric parameters (Å, °)

**Table:**

Co1—O2W	2.0559 (18)	C2—C7	1.385 (4)
Co1—O2W^i^	2.0559 (18)	C2—C3	1.395 (4)
Co1—O1W^i^	2.094 (2)	C3—C4	1.383 (4)
Co1—O1W	2.094 (2)	C3—H3	0.9300
Co1—O3W^i^	2.141 (2)	C4—C5	1.384 (4)
Co1—O3W	2.141 (2)	C4—H4	0.9300
O1—C1	1.254 (3)	C5—C6	1.387 (4)
O2—C1	1.268 (3)	C6—C7	1.387 (4)
O3—C5	1.372 (3)	C6—H6	0.9300
O3—C8	1.420 (3)	C7—H7	0.9300
O1W—H11	0.837 (10)	C8—C9	1.507 (4)
O1W—H12	0.838 (10)	C8—H8A	0.9700
O2W—H21	0.841 (10)	C8—H8B	0.9700
O2W—H22	0.841 (10)	C9—C13	1.381 (4)
O3W—H31	0.847 (10)	C10—C11	1.372 (5)
O3W—H32	0.846 (10)	C10—H10	0.9300
O4W—H41	0.837 (10)	C11—C12	1.369 (5)
O4W—H42	0.839 (10)	C11—H11A	0.9300
N1—C9	1.334 (4)	C12—C13	1.386 (4)
N1—C10	1.344 (4)	C12—H12A	0.9300
C1—C2	1.497 (4)	C13—H13	0.9300
			
O2W—Co1—O2W^i^	180.000 (1)	C2—C3—H3	119.8
O2W—Co1—O1W^i^	93.59 (8)	C3—C4—C5	119.9 (3)
O2W^i^—Co1—O1W^i^	86.41 (8)	C3—C4—H4	120.1
O2W—Co1—O1W	86.41 (8)	C5—C4—H4	120.1
O2W^i^—Co1—O1W	93.59 (8)	O3—C5—C4	114.6 (2)
O1W^i^—Co1—O1W	180.0	O3—C5—C6	124.8 (3)
O2W—Co1—O3W^i^	86.46 (8)	C4—C5—C6	120.5 (2)
O2W^i^—Co1—O3W^i^	93.54 (8)	C5—C6—C7	119.0 (3)
O1W^i^—Co1—O3W^i^	92.45 (9)	C5—C6—H6	120.5
O1W—Co1—O3W^i^	87.55 (8)	C7—C6—H6	120.5
O2W—Co1—O3W	93.54 (8)	C2—C7—C6	121.3 (3)
O2W^i^—Co1—O3W	86.46 (8)	C2—C7—H7	119.3
O1W^i^—Co1—O3W	87.55 (8)	C6—C7—H7	119.3
O1W—Co1—O3W	92.45 (9)	O3—C8—C9	107.6 (2)
O3W^i^—Co1—O3W	180.000 (1)	O3—C8—H8A	110.2
C5—O3—C8	119.4 (2)	C9—C8—H8A	110.2
Co1—O1W—H11	106 (3)	O3—C8—H8B	110.2
Co1—O1W—H12	117 (3)	C9—C8—H8B	110.2
H11—O1W—H12	109.7 (17)	H8A—C8—H8B	108.5
Co1—O2W—H21	117 (2)	N1—C9—C13	122.4 (3)
Co1—O2W—H22	130 (2)	N1—C9—C8	115.3 (2)
H21—O2W—H22	109.4 (16)	C13—C9—C8	122.3 (2)
Co1—O3W—H31	104 (3)	N1—C10—C11	123.2 (3)
Co1—O3W—H32	117 (3)	N1—C10—H10	118.4
H31—O3W—H32	107.6 (16)	C11—C10—H10	118.4
H41—O4W—H42	109 (6)	C12—C11—C10	118.7 (3)
C9—N1—C10	117.8 (3)	C12—C11—H11A	120.7
O1—C1—O2	123.3 (2)	C10—C11—H11A	120.7
O1—C1—C2	119.1 (2)	C11—C12—C13	119.1 (3)
O2—C1—C2	117.6 (2)	C11—C12—H12A	120.4
C7—C2—C3	118.8 (2)	C13—C12—H12A	120.4
C7—C2—C1	120.9 (2)	C9—C13—C12	118.8 (3)
C3—C2—C1	120.4 (2)	C9—C13—H13	120.6
C4—C3—C2	120.5 (3)	C12—C13—H13	120.6
C4—C3—H3	119.8		
			
O1—C1—C2—C7	3.0 (4)	C1—C2—C7—C6	−179.5 (3)
O2—C1—C2—C7	−175.9 (3)	C5—C6—C7—C2	0.6 (5)
O1—C1—C2—C3	−176.1 (3)	C5—O3—C8—C9	176.6 (3)
O2—C1—C2—C3	5.1 (4)	C10—N1—C9—C13	−0.8 (5)
C7—C2—C3—C4	0.1 (5)	C10—N1—C9—C8	177.9 (3)
C1—C2—C3—C4	179.2 (3)	O3—C8—C9—N1	178.4 (3)
C2—C3—C4—C5	0.1 (5)	O3—C8—C9—C13	−2.9 (4)
C8—O3—C5—C4	−177.1 (3)	C9—N1—C10—C11	0.1 (5)
C8—O3—C5—C6	2.2 (5)	N1—C10—C11—C12	0.3 (6)
C3—C4—C5—O3	179.3 (3)	C10—C11—C12—C13	0.0 (6)
C3—C4—C5—C6	0.1 (5)	N1—C9—C13—C12	1.1 (5)
O3—C5—C6—C7	−179.6 (3)	C8—C9—C13—C12	−177.6 (3)
C4—C5—C6—C7	−0.4 (5)	C11—C12—C13—C9	−0.6 (5)
C3—C2—C7—C6	−0.5 (5)		

Symmetry codes: (i) −*x*+1, −*y*+1, −*z*+1.

### Hydrogen-bond geometry (Å, °)

**Table:**

*D*—H···*A*	*D*—H	H···*A*	*D*···*A*	*D*—H···*A*
O1w—H11···O1	0.84 (1)	1.94 (1)	2.762 (3)	169 (3)
O1w—H12···O4w^ii^	0.84 (1)	1.87 (1)	2.696 (4)	171 (4)
O2w—H21···O2	0.84 (1)	1.84 (1)	2.678 (3)	179 (3)
O2w—H22···O2^iii^	0.84 (1)	1.97 (2)	2.755 (3)	156 (3)
O3w—H31···O1^i^	0.85 (1)	1.96 (1)	2.798 (3)	170 (4)
O3w—H32···N1^iv^	0.85 (1)	1.95 (1)	2.799 (3)	177 (4)
O4w—H41···O2	0.84 (1)	2.22 (4)	2.891 (4)	137 (5)
O4w—H42···O3w^v^	0.84 (1)	2.42 (4)	3.176 (4)	151 (7)

Symmetry codes: (ii) −*x*, −*y*+2, −*z*+1; (iii) −*x*+1, −*y*+2, −*z*+1; (i) −*x*+1, −*y*+1, −*z*+1; (iv) *x*, *y*−1, *z*+1; (v) *x*, *y*+1, *z*.
